# Quality of Life and Psychosocial Impact of Sport on People with Intellectual Disabilities: The Value of First-Person Discourse

**DOI:** 10.3390/healthcare14183089

**Published:** 2026-09-19

**Authors:** Oscar Martínez-Rivera, Cristina Alonso-López, Jordi Planella

**Affiliations:** 1 Blanquerna, Universitat Ramon Llull, 08001 Barcelona, Spain; 2 Faculty of Educational Studies., Universitat Oberta de Catalunya, 08018 Barcelona, Spain

**Keywords:** intellectual disabilities, quality of life, sport, physical activity, people with disabilities

## Abstract

**Background:** The concept of quality of life (QoL) has become a fundamental focus of research involving people with intellectual disabilities (IDs). Numerous studies have explored this concept. Furthermore, incorporating the first-person perspectives of people with disabilities has become essential in contemporary research. However, no studies have rigorously examined how sport participation among people with IDs is reflected across the QoL dimensions proposed by Schalock and Verdugo. The published literature on these aspects also suggests other emerging topics analyzed. **Methods:** A thematic synthesis was applied, structured around a directed qualitative content analysis of direct quotes from people with intellectual disabilities published in scientific articles, modified according to the dimensions of Quality of Life and identifying emerging categories. Subsequently, the codes were grouped into descriptive themes that were reinterpreted to generate higher-order analytical themes. A total of 390 direct quotes were analyzed from 24 selected articles. **Results:** The published accounts of the participants associated sports participation with several aspects of Quality of Life that engaging in sports provides them. The opinions of the individuals themselves refer to all areas of quality of life. Statements were also found referring to aspects such as having an occupation or a routine, other experiences (beyond strictly sporting ones), transition to adulthood, and negative statements and barriers. **Conclusions:** The testimonies of people with intellectual disabilities show that sport transcends the physical domain, impacting various dimensions of their quality of life. They report that sports practice depends on opportunities, support, and environmental barriers.

## 1. Introduction

Sport participation among people with intellectual disabilities (IDs) has a positive impact on physical health, and extensive scientific evidence demonstrates its cardiovascular and musculoskeletal benefits [1]. Evidence also points to the financial burden and the increased use of healthcare services resulting from physical inactivity [2,3]. By contrast, fewer studies have examined the psychosocial impact of sport participation. In this regard, the benefits of sport extend far beyond physical health [4,5,6].

A smaller body of research has specifically examined the impact of sport on QoL, although the available evidence consistently reports benefits across all QoL dimensions [7,8,9]. Given that QoL is a central concept in research involving people with IDs, further investigation into the relationship between sport participation and QoL is warranted.

However, most studies conducted in this field have not incorporated the first-person perspectives of people with disabilities. Research has typically relied on the views of other stakeholders, such as family members or coaches, or has been conducted within a quantitative paradigm without directly asking the individuals concerned about their own experiences.

This research aims to understand, from the perspective of people with intellectual disabilities (IDs), how the experience of sports practice relates to the different dimensions of their quality of life (QoL), based on a synthesis of their first-person testimonies gathered in previous studies, as well as to identify other emerging themes within their discourse.

The methodology employed was based on the thematic synthesis method proposed by Thomas and Harden (2008), featuring an initial phase of directed qualitative content analysis [10]. Subsequently, an analytical process was conducted to identify emerging categories. The entire process was applied exclusively to direct quotes from individuals with IDs in scientific articles. Coding was performed using the dimensions of Schalock and Verdugo’s QoL model (2005, 2010, 2021) as a framework, alongside the emerging categories [11,12,13,14]. Subsequently, the codes were grouped into descriptive themes, which were then reinterpreted to generate higher-order analytical themes integrating the findings across all included studies. Prior to this process, scientific publications meeting the inclusion criteria for this research were identified through a PRISMA-guided selection process.

### 1.1. Positive Impact of Sport on People with Intellectual Disabilities

Although the available evidence consistently demonstrates the positive impact of sport participation, people with disabilities remain less likely to achieve the internationally recommended levels of physical activity [15]. They are twice as likely to be physically inactive [16], and research indicates that between 16% and 62% of people with disabilities are less likely to meet these international recommendations. This situation may be even more pronounced among people with IDs [17], despite their willingness to participate in sporting activities [18]. Furthermore, countries experiencing higher levels of poverty rarely report data on sport participation [19]. This situation poses a significant risk to both health and QoL [20]. Consequently, the United Nations promotes access to leisure and sporting activities through the Convention on the Rights of Persons with Disabilities [21,22].

It is widely recognised that the health benefits of physical activity extend beyond physical health and include important psychosocial benefits [23]. In this regard, sport participation contributes to improvements in interpersonal relationships and community participation, facilitating the development of new friendships and fostering a sense of belonging [24,25,26]. In this sense, McConkey et al. (2019) [24] frame sport as a vehicle with potential to promote the social inclusion of individuals with intellectual disabilities, especially when facilitating shared participation between people with and without disabilities. From the participants’ own perspective, inclusion extends beyond physical presence in athletic activities, centering on a sense of togetherness characterized by equality, friendship, active involvement, interpersonal bonds, and mutual assistance. Additionally, this experience was linked to feelings of happiness, relaxation, self-confidence, and a sense of being cared for.

Varahra et al. (2022) [5] point out that the development during adolescence of skills related to mobility, social competences, and independent self-care can influence subsequent educational and employment participation in adulthood. In this context, the authors highlight the value of incorporating exercise and sport into programmes aimed at adolescents and young people with disabilities, considering their potential relevance for planning the transition to adulthood. Improvements have also been reported in daily living skills and leadership abilities [27], together with the development of self-esteem, self-confidence, and resilience, all of which are closely associated with goal attainment [17,23]. These benefits are closely related to the organisation of sporting events for people with IDs, which promote far more than athletic performance alone. In this respect, positive outcomes extend beyond the athletes themselves to influence their wider social environment [28] and may even contribute to changing societal attitudes and beliefs towards people with IDs [25].

Adopting a rights-based framework, Kiuppis (2018) [29] argues for an understanding of sports inclusion that moves beyond simple access, drawing attention to the terms and conditions of participation for people with disabilities. Consequently, he recognizes the spectrum of participation pathways while emphasizing personal preferences and choices. This perspective frames individuals with disabilities as active agents in their sports involvement, conceptualising inclusion as a dynamic process that must account for both environmental opportunities and self-determined decisions.

Nevertheless, substantial evidence indicates that people with disabilities continue to face significant barriers to exercising their right to social participation. These limitations are not restricted to sport but also affect leisure activities and other areas of daily life. Such restrictions are largely associated with attitudinal and institutional barriers linked to ableism. Universal accessibility, public policies, and personal assistance have all been identified as key facilitators for increasing participation across all areas of life for people with disabilities [30].

These challenges extend directly to the participation of people with IDs in physical activity and sport. The literature identifies systemic barriers including transportation issues, facility access, a scarcity of adapted programmes, the requirement for dedicated support or accompaniment, social integration difficulties, and negative experiences across specific sports settings. Such constraints restrict opportunities for engagement, underscoring that acknowledging the right to sport does not automatically guarantee its practical implementation [25,31,32]. Under these conditions, simply having the opportunity to participate in a meaningful activity can yield positive outcomes for individuals with IDs [33].

In response to these circumstances, the Special Olympics movement was established in the early 1960s to address both the social and physical needs of this population. Since then, the organisation has achieved a positive international impact on the physical health, psychosocial well-being, and QoL of people with IDs [34,35].

Armila et al. (2018) [36] address sport from a leisure perspective and as a way for young people with intellectual disabilities to spend their free time. From this perspective, sports practice transcends its physical and rehabilitative dimension and gains relevance due to the opportunities it offers to interact with peers, share experiences, and have fun. Once again, the possibilities of enjoying sport during free time can be conditioned by factors such as the availability of suitable activities, distances and transport, the presence of trained coaches, and support networks.

Finally, it is worth highlighting the role of coaches and support professionals as relevant figures in facilitating and sustaining the sports participation of people with IDs. Coaches can perform functions that go beyond sports teaching, fostering the development of skills, meaningful relationships, and the adaptation of practice to individual needs [37]. Likewise, support staff can contribute through communication, structuring the activity, motivation, and necessary adaptations [38]. In this sense, the capabilities, opportunities, and motivations of these professionals can act as facilitators or barriers to the support provided and, therefore, condition the opportunities for participation in physical activity for people with IDs [39].

Strong scientific evidence also supports the psychosocial benefits of sport participation [40,41,42], particularly regarding improvements in the QoL of people with IDs [43,44].

### 1.2. Quality of Life of People with Intellectual Disabilities

The concept of QoL among people with IDs has become one of the most extensively researched topics in recent decades. The social model of disability has transformed the understanding of human diversity, highlighting the importance of constructs such as QoL and shifting attention beyond purely medical and rehabilitative perspectives. Despite the international recognition of the rights of people with IDs, research consistently demonstrates a substantial gap in QoL between people with IDs and the general population. Consequently, QoL has become a key reference framework for research in this field [45,46].

Schalock and Verdugo are among the leading scholars in this area, having transformed QoL into an operational and measurable construct through decades of research [47,48]. Their work has led to the development of assessment instruments capable of measuring the QoL of people with disabilities. This represents an important step towards the design of public policies that promote substantial improvements in the lives of people with IDs. Such policies should be based on supports and services that enhance people’s QoL [14,49].

Furthermore, the Schalock and Verdugo QoL model has also enabled the assessment of QoL among people with severe IDs, a population that has not always been adequately represented in other models [50]. In addition, the construct has demonstrated cross-cultural validity [51,52].

QoL has been defined as a multidimensional construct comprising eight core dimensions: self-determination (SD), rights (RI), Emotional Well-being (EW), social inclusion (SI), personal development (PD), interpersonal relationships (IR), material well-being (MW), and physical well-being (PW) [47].

These dimensions are clearly embedded within the social model of disability, in which the surrounding environment plays a fundamental role and directly influences QoL. All dimensions have been widely examined in international research, with self-determination recognised as a central dimension closely related to all the others. For example, people living in environments where their rights are recognised are more likely to experience higher levels of self-determination [53].

### 1.3. Inclusive Methodologies Incorporating First-Person Perspectives

Despite growing efforts to incorporate the first-person perspectives of people with IDs into research, relatively few studies have done so. This reflects the broader challenge that society still faces in ensuring the meaningful participation of people with disabilities across all areas of life.

Failing to include the voices of those who are the focus of research has important scientific implications, as it constitutes a clear example of epistemic injustice, whereby vulnerable groups are either not heard, or their knowledge is not afforded the social value it deserves [54].

The present study addresses this gap by focusing specifically on the voices of people with IDs themselves. Most existing studies discuss the experiences of people with disabilities through the perspectives of others. Although the views of family members, coaches, and other significant stakeholders are undoubtedly valuable in sport-related research, they cannot replace the direct accounts of people with disabilities.

Studies that seek to incorporate first-person perspectives should include measures that ensure cognitive accessibility and facilitate participants’ understanding of the research process. Using easy-to-understand language, involving trusted support persons when appropriate, and adapting the complexity of interview questions are among the strategies most frequently recommended in the literature to maximise the quality of participants’ responses [55].

More recent research demonstrates increasing methodological awareness of these issues and proposes strategies that enable people with IDs to understand research procedures and participate meaningfully [56]. Although such studies remain relatively uncommon, many researchers now make considerable efforts to include the direct perspectives of people with disabilities, even in situations where participants experience severe communication difficulties or are non-speaking [57].

Research has also begun to include people with IDs not only as participants but as members of research teams, enabling them to contribute to decision-making throughout the different stages of the research process [58]. Nevertheless, publications that provide rigorous methodological guidance on how to conduct genuinely inclusive and cognitively accessible research remain scarce [59].

Individual interviews were considered the most appropriate method for obtaining the first-person accounts required to address the objectives of this study. Although focus groups can generate valuable complementary information, they may also introduce bias through the development of group consensus or responses influenced by social desirability [60]. Furthermore, individual interviews are particularly suitable for exploring personal experiences related to participants’ self-assessments of the different QoL dimensions [61].

## 2. Materials and Methods

This study was conducted by analysing first-person statements made by people with IDs reported in scientific publications. These statements, extracted from the results sections of the included articles, were analysed using the eight dimensions of the QoL model proposed by Schalock and Verdugo (2005, 2010, 2021) within the framework of thematic synthesis [11,12,13,14].

The QoL model developed by Schalock and Verdugo (2005, 2010, 2021) [11,12,13,14] served as a theoretically informed analytical framework that guided the initial coding process and the organisation of the findings. At the same time, the comparative and interpretative analysis across studies enabled the development of new conceptual relationships and higher-order interpretations consistent with the principles of qualitative metasynthesis. Accordingly, the model was not used as a rigid classification system but rather as a conceptual framework that facilitated the identification and interpretation of meanings embedded in participants’ accounts, while maintaining a continuous dialogue between the empirical evidence and a well-established theoretical framework in the field of QoL. In parallel, the analytical process enabled the identification of emergent categories that were not fully captured within the pre-established dimensions of the model, which contributed to nuancing and broadening the understanding of the psychosocial impact attributed to sports practice by people with intellectual disabilities.

The data analysis combined a deductive and inductive approach. In the first phase, a directed qualitative content analysis was conducted [62], using the eight quality of life (QoL) dimensions proposed by Schalock and Verdugo (2005, 2010, 2021) [11,12,13,14] as a deductive framework. This framework guided the initial coding of first-person quotations extracted from the included studies, whilst maintaining an open approach to those contents not sufficiently represented by the a priori established categories, which allowed the identification of emergent themes. In a second phase, the coded data were integrated through a thematic synthesis, following the logic proposed by Thomas and Harden (2008) [10]. Based on line-by-line coding, conceptually related contents were grouped into descriptive themes, endeavouring to keep them close to the experiences expressed by the people with IDs themselves. Subsequently, analytical themes were developed through a cross-cutting interpretation of the patterns identified across the studies, moving beyond the individual description of quotations. This process enabled the generation of an interpretive synthesis of each QoL dimension and the emergent themes, preserving the perspective of people with IDs and avoiding attributing causal relationships or generalisations to their testimonies that exceeded the scope of the included studies.

Regarding reflexivity, all three authors have extensive experience working with people with intellectual disabilities and adopt a position on disability based on human rights and the Convention on the Rights of Persons with Disabilities [21], identifying with independent living movements. The first author has been a Special Olympics coach and volunteer.

### 2.1. Search Strategy and Study Selection

The literature search was conducted in the Scopus and Web of Science (WoS) and was complemented with EBSCO Discovery Service, as a supplementary source, to maximise the volume of relevant literature.

An initial search was conducted on 1 June 2026 using the Boolean equation: “intellectual disability” AND “sport” AND “interview” AND “adult”. Subsequently, the search was broadened using further keywords to maximise the volume of relevant literature. On 4 August 2026, the search was expanded using the Boolean equation: (“Intellectual disability” OR “developmental disability” OR “learning disability”) AND (Sport OR exercise OR “physical activity”) AND (Interview OR narrative OR “focus group*” OR “qualitative research” OR “lived experience”) AND adult*.

To ensure that the evidence included was both current and of high scientific quality, the following eligibility criteria were applied:

- Publication period: 2016–2026.
- Document type: peer-reviewed scientific articles.
- Language: English.

**Figure 1:**
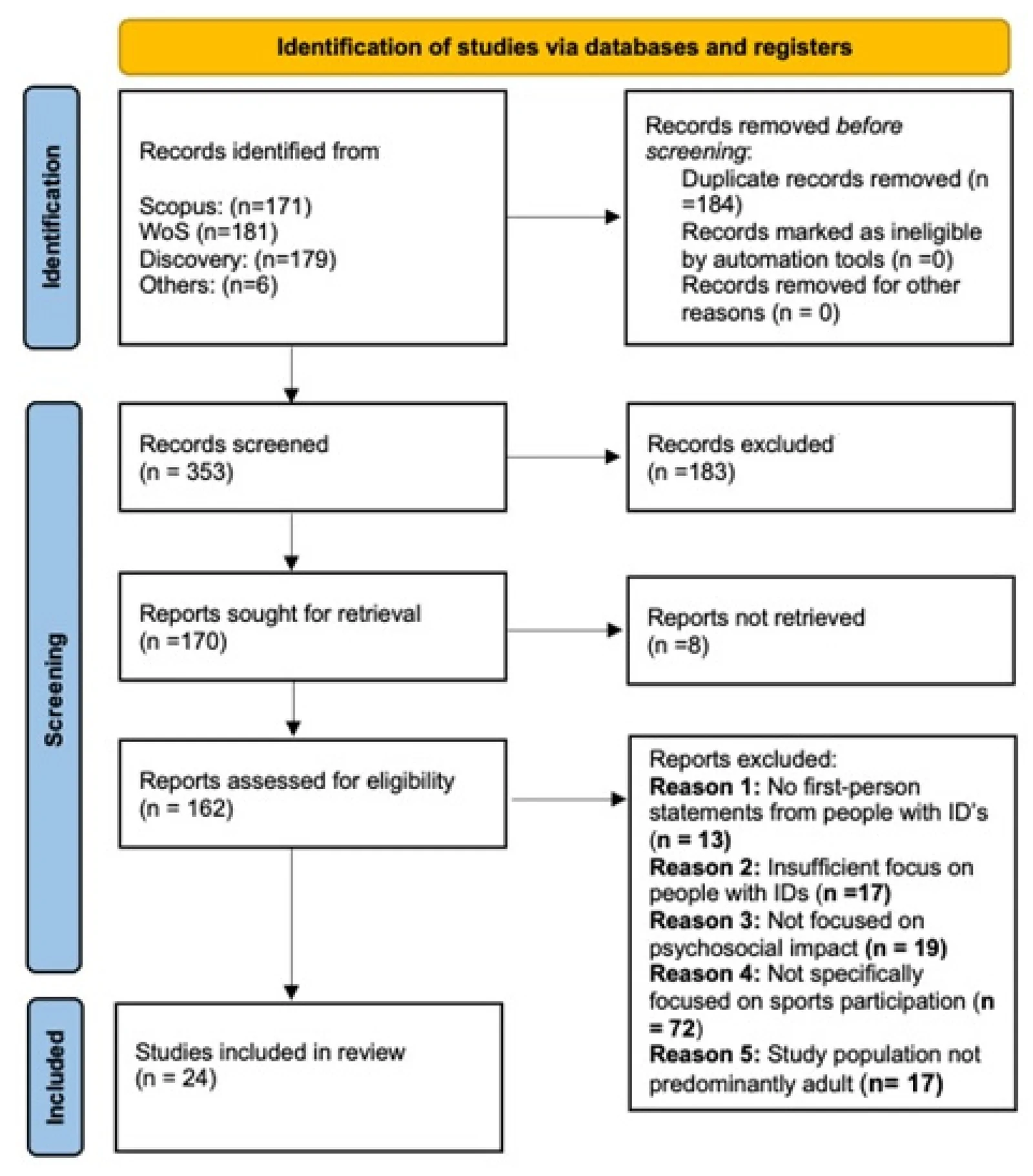
PRISMA flow diagram illustrating the identification, screening, eligibility assessment, and inclusion of studies.

The identification, screening, and selection of studies followed the PRISMA 2020 guidelines, as illustrated in the flow diagram below (Figure 1).

The study selection process was carried out following the different phases set out in the PRISMA diagram. In the identification phase, a total of 537 records were located, originating from Scopus (*n* = 171), Web of Science (*n* = 181), Discovery (*n* = 179), and other sources (*n* = 6). Prior to screening, 184 duplicate records were removed, with no exclusions made using automated tools or for other reasons. As a result, 353 records progressed to the screening phase. During screening, 183 records were excluded, leaving 170 publications for retrieval. Of these, eight could not be retrieved, meaning 162 publications were assessed at full text for eligibility.

Following full-text evaluation, 138 publications were excluded. The primary reason for exclusion was that the studies were not specifically focused on sports participation (*n* = 72). Likewise, studies were excluded if they did not analyse psychosocial impact (*n* = 19), if their population was not predominantly adult (*n* = 17), if people with intellectual disabilities were insufficiently central to the study (*n* = 17), and if they did not include first-person statements from people with intellectual disabilities (*n* = 13). Ultimately, 24 studies met the established criteria and were included in the review.

Regarding this selection, in the case of the articles by Elipe-Lorenz et al. (2026) [63] and Corazza (2017) [64], the interviews involved both people with IDs and people without this characteristic. The articles made it possible to isolate the first-person statements of people with IDs, and only the latter were analysed. With reference to the articles by Maenhout et al. (2026) [65] and Kim et al. (2025) [66], it was decided to include them because each article only incorporates two adolescents and predominantly includes adults. Furthermore, in the second case, the authors specify that the inclusion of two individuals under the age of 18 was an exception, and the research was designed for adults. The selected studies included in the analysis are described in Table 1.

The selection criteria were agreed upon by the three researchers. The first author conducted an initial review, highlighting articles that raised doubts. The final selection was made based on independent judgement, followed by consensus among the three researchers. Further details on search strategies and eligibility criteria can be found in File S1.

To assess the methodological quality of the included studies, the Critical Appraisal Skills Programme (CASP) [87] was used. CASP is one of the most widely employed instruments for the critical appraisal of qualitative research because it comprehensively addresses the principal quality criteria and methodological assumptions underpinning qualitative inquiry (Table 2). The quality appraisal was conducted to describe the methodological rigour of the included studies and to inform the synthesis process. Consequently, no study was excluded on the basis of methodological quality, as the aim of the metasynthesis was to integrate all qualitative evidence that met the predefined inclusion criteria.

Although some of the included studies did not meet criterion 6 of the CASP tool, regarding the consideration of the relationship between researcher and participants, this aspect was not established as an exclusion criterion. Limited reflexivity makes it difficult to assess the extent to which the researcher’s position, their relationship with participants, or the interview context may have influenced the testimonies obtained. Assessment using CASP was employed to overall evaluate the methodological quality of the studies and identify potential limitations, rather than to automatically exclude research due to non-compliance with an individual criterion. Nevertheless, the included articles demonstrated adequate compliance with the remaining methodological criteria deemed essential to address the review’s objective; therefore, it was considered that this limitation did not sufficiently compromise their overall quality to warrant exclusion. Furthermore, the origin of the testimonies supporting the main analytical themes was examined in relation to the CASP assessment of the primary studies. This procedure made it possible to verify whether certain findings depended exclusively on studies with greater methodological limitations or whether they were supported by studies with varying quality levels.

The assessment of methodological quality using CASP was also incorporated into the process of synthesising the results. When an analytical theme included testimonies from studies whose criterion 6 of CASP had been assessed as not met or partially met, an effort was made, whenever possible, to incorporate an additional testimony from a study assessed as totally met for this criterion. When a theme did not include testimonies from studies that fully met this criterion, this circumstance was explicitly indicated by means of a note below the corresponding table.

On the other hand, one of the studies was classified as “Not met” on CASP criterion 7, regarding the consideration of ethical issues. This rating was due to the article lacking explicit information regarding approval by an ethics committee, the informed consent procedure, or the measures taken to ensure participant confidentiality and anonymity. Considering its overall characteristics, it was decided to include it.

### 2.2. Data Analysis

The unit of analysis consisted of the verbatim quotations provided by people with IDs reported in the twenty-four scientific publications included in the review.

To foster the consistency of the analytical process, coding was developed through a sequential procedure of consensus and review among the three researchers. First, all three researchers conducted an initial reading of the included studies to familiarise themselves with their content and the themes addressed. The first five articles were analysed jointly, which allowed the coding criteria to be agreed upon and a common procedure for the analysis of the remaining studies to be established. Based on these criteria, the second researcher carried out line-by-line coding of 365 first-person quotations from people with intellectual disabilities (IDs) using ATLAS.ti software, version 26. During this process, quotations were classified according to the eight quality of life (QoL) dimensions proposed by Schalock and Verdugo (2005, 2010, 2021) [11,12,13,14], whilst fourteen emerging themes derived from the data were identified. Subsequently, the principal investigator reviewed the coding and added 25 quotations that met the previously agreed criteria, reaching a total of 390 quotations. Finally, the third researcher reviewed the entire coding and the results obtained, with no relevant discrepancies identified. In this way, the analysis process was supported by criteria shared and agreed upon by all three researchers. Multiple coding was allowed.

For the general analysis of the results, the frequency of codes associated with each category was examined descriptively to show their presence and recurrence across the different analysed sources, without drawing inferences regarding their relative relevance or importance.

The qualitative synthesis followed the thematic synthesis method proposed by Thomas and Harden (2008) [10], using exclusively the verbatim quotations from people with intellectual disabilities reported in the results section of the selected studies. Coding was undertaken using the domains of Schalock and Verdugo’s QoL model as the analytical framework [11,12,13,14]. The codes were subsequently grouped into descriptive themes according to their conceptual similarities and were finally reinterpreted to generate higher-order analytical themes that integrated the findings across all included studies.

All data were managed using ATLAS.ti, which facilitated the organisation and systematic management of the analytical process. Throughout the analysis, analytical memos were produced to document methodological decisions, interpretative reflections, and the emerging relationships between categories. The final synthesis was organised into a summary table presenting, for each QoL dimension, the descriptive themes, the most representative quotations, and the corresponding higher-order analytical themes.

All analyzed textual quotes can be found in Table S1.

## 3. Results

### 3.1. First Level: Analysis of Results by Frequency

**Figure 2:**
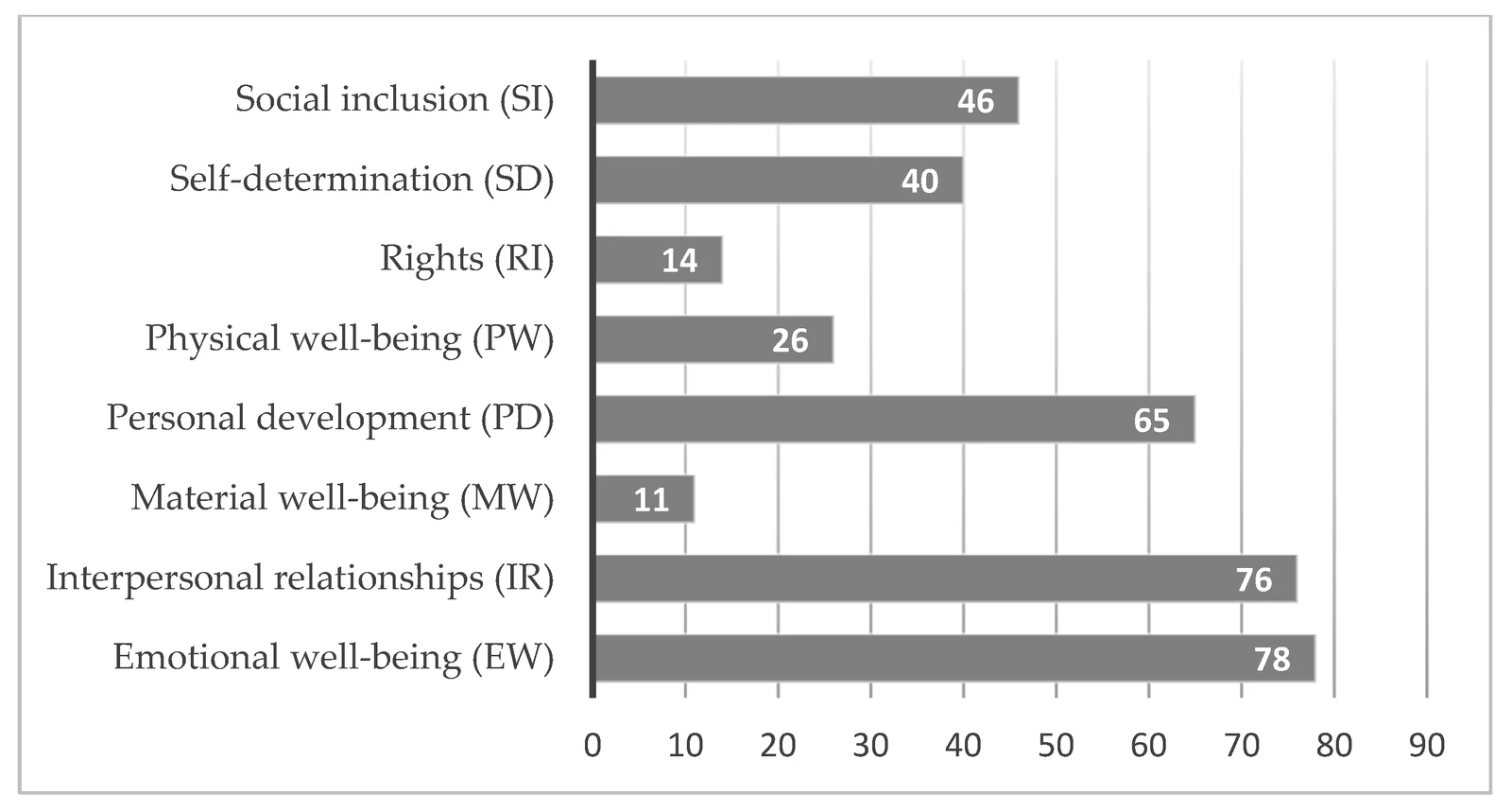
Results by frequency.

The verbatim quotations extracted from the included studies yielded an initial distribution of codes across the QoL dimensions proposed by Schalock and Verdugo (Figure 2).

The descriptive frequency analysis shows differences in the number of quotations coded across the eight quality of life dimensions. Emotional well-being (EW) registers 78 quotations and interpersonal relations (IR), 76. These are followed by personal development (PD), with 65 quotations; social inclusion (SI), with 46; and self-determination (SD), with 40. The frequencies recorded for physical well-being (PW), rights (RI), and material well-being (MW) are 26, 14, and 11 quotations, respectively. These values represent exclusively the frequency with which quotations from the analysed corpus were coded under each dimension and do not imply differences in their importance, relevance, or weight within quality of life.

### 3.2. Thematic Synthesis

#### 3.2.1. Quality of Life Thematic Synthesis

The results of the thematic synthesis are presented below, organized according to the dimensions of the QoL model.

In Table 3, you can read about thematic synthesis of Emotional Well-being (EW).

Overall, the analytical themes suggest that, from the perspective of the people with IDs participating in the included studies, sports practice can represent a significant context for their emotional well-being. Their experiences point to a combination of enjoyment, connections and belonging, confidence in their own capabilities, growth, and personal appreciation, alongside opportunities to cope with negative emotional states and feel safe to express their emotions. Furthermore, some accounts indicate that environmental conditions, including contact with outdoor and natural spaces, could contribute to this experience. In this sense, emotional well-being emerges as a multidimensional experience in which personal, relational, and contextual aspects converge, strictly based on the perceptions expressed by the participants themselves and without these results allowing for the establishment of causal or generalisable relationships.

In Table 4, you can read about thematic synthesis of interpersonal relationships (IR).

Overall, the analytical themes suggest that, from the perspective of the people with IDs participating in the included studies, sport can constitute a relevant space for building and consolidating interpersonal relations. Their experiences point to the possibility of expanding social contacts and developing friendships that can transcend the sports context itself, generating bonds characterised by companionship, support, and closeness. These relationships do not appear to be limited to peers, but can also include coaches and instructors as significant figures, while the team can acquire a sense of unity and family. Likewise, the accounts reflect dynamics of reciprocity, recognition, mutual aid, and shared enjoyment, alongside opportunities to interact and communicate with others.

In Table 5, you can read about thematic synthesis of personal development (PD).

On the whole, the analytical themes suggest that, from the perspective of the people with IDs participating in the included studies, sport can represent a context for learning and the progressive acquisition of skills, accompanied by a perception of growth and increased confidence in their own capabilities. This development also appears to be expressed through the recognition of personal achievements, overcoming previously perceived challenges and limits, and setting new goals and aspirations. In turn, some accounts point towards a progressive assumption of responsibilities and roles of leadership and support for others, as well as learnings that can be projected onto communication and social interaction. In this sense, personal development emerges in the discourses as a process that could go beyond the improvement of sports skills, incorporating experiences of progress, autonomy, challenge, and active participation in relation to others.

In Table 6, you can read about thematic synthesis of physical well-being (PW).

Regarding physical well-being, the discourses of people with IDs participating in the included studies reflect a favourable perception of sports practice and physical activity in relation to their health, physical condition, and functional capacity. Some accounts point to feeling stronger and fitter to cope with daily life activities, whilst others incorporate goals linked to weight and their own bodies. The association of physical activity with maintaining more active routines and a greater presence of movement in daily life is also observed. Thus, based on the experience expressed by the participants themselves, this dimension appears to encompass not only the perception of health, but also physical capacity and the maintenance of an active lifestyle.

In Table 7, you can read about thematic synthesis of Rights (RI).

In the rights dimension, the accounts of people with IDs participating in the included studies position sport as a domain related to access to opportunities and the possibility of participating on equal terms. Their experiences reflect both situations characterised by respect and the absence of discrimination, as well as others in which participation may be limited by decisions or barriers linked to disability. Faced with these situations, some discourses highlight the relevance of having the necessary supports to be able to participate. From the perspective of the participants themselves, rights in the sports context thus appear to be articulated around three closely related elements: opportunity for participation, equal treatment, and the availability of adequate supports.

In Table 8, you can read about thematic synthesis of material well-being (MW).

The material well-being dimension shows different ways in which material conditions can intertwine with the sports experience. From the perspective of people with IDs, economic and employment constraints that can limit dedication to sport appear on the one hand, and opportunities associated with participation—such as access to employment, travel, and other experiences outside the usual environment—appear on the other. Tangible or symbolic forms of recognition linked to sporting achievements are also identified. Thus, the accounts suggest a two-way relationship: material circumstances can condition sports participation, while participation can open up access to certain opportunities, experiences, and recognitions.

In Table 9, you can read about thematic synthesis of self-determination (SD).

In the self-determination dimension, the discourses of people with IDs highlight sport as a context in which they can exercise choice, make decisions, and develop greater independence, both individually and alongside others. This capacity to act upon their own participation is also linked to defining and pursuing personal goals, accompanied by attitudes of effort and persistence in the face of difficulties. Furthermore, some accounts reflect the opportunity to take on responsibilities and adopt an active role within sports activities. From the participants’ perspective, self-determination thus appears to integrate the ability to decide on their own experience, direct actions towards goals, and assume commitments and responsibilities.

In Table 10, you can read about thematic synthesis of Social Inclusion (SI).

Social inclusion is reflected in the discourses of people with IDs primarily through the possibility of feeling part of a group and recognized as members of a community. This experience appears to be constructed through shared participation, which fosters contact and interaction with others, as well as active involvement in collective dynamics. From the participants’ perspective, inclusion linked to sport thus appears to integrate belonging, social connection, and opportunities to be part of and participate in a shared space.

#### 3.2.2. Thematic Synthesis of Emerging Themes

During the analysis process, 14 emerging themes were identified: having a supportive role, having an occupation and routine, helping and teaching others, humour, athletic identity, motivation, contact with nature and fresh air, negative statements, other experiences, positive coping, reward and recognition, self-esteem, sense of belonging, and transition to adulthood. Some of these themes were subsequently integrated into the dimensions of the QoL model, as they matched the themes developed in the thematic synthesis.

Among the integrated themes, four demonstrated a cross-cutting nature, as they were linked to more than one quality of life dimension: helping and teaching others was related to Personal Development and Interpersonal Relationships; reward and recognition to Material Well-Being and Personal Development; self-esteem to Emotional Well-Being and Personal Development; and sense of belonging to Social Inclusion and Interpersonal Relationships.

On the other hand, having an occupation or a routine, other experiences, transition to adulthood, and negative statements and barriers were not integrated as specific themes within the QoL dimensions. Finally, having a supportive role, although reflected in content related to the Interpersonal Relationships dimension, was addressed as an independent theme given its relevance.

##### Having an Occupation or a Routine

The emerging theme having an occupation reflects the importance that participants attach to having activities to carry out and staying busy. This idea appears explicitly when a good life is associated with having occupations: “A good life is having occupations, that is. Having things to do” [74]. In this sense, sports practice can constitute a concrete form of occupation, as expressed by a participant noting that “[i]t just also gave me something to do” [66].

The testimonies also show a contrast between staying busy and inactivity. Kapsalakis et al. (2024) [74] document how the availability of activities is valued positively against boredom: “I like helping out when there’s something to do; when there’s work, it’s good! But when there’s nothing to do and we just sit around, it’s boring.” This same contrast appears directly linked to sports activity in the statement: “It [walking programme] means that I am not lying in bed half of the morning and I am also walking for the sake of it” [77].

Likewise, sports practice appears linked to time management and the incorporation of regular activities into daily life. In some cases, this organization is externally structured through established schedules, as one participant points out: “We do get a monthly schedule. With the time, place and the coach in charge, and their phone number” [79]. In other accounts, physical activity forms part of a habitual routine, as shown by the statement: “I’d go to the gym 3 times a week. And that would keep me active. I’d still go to that and I’d still do my walks in the mornings around town” [68]. Specific schedules are also described where activity is combined with other daily obligations, such as work: “And half past five walking starts. (…) Then I’m still tired. Because I was eight hours at work” [76].

##### Other Experiences

The emerging theme other experiences primarily captures experiences linked to travel, trips, and opportunities to visit new places associated with sports participation. In some testimonies, travelling is in itself a valued part of the experience, as expressed by one participant: “The best part is travelling to competitions. The happiness from travelling.” [81]. Similarly, Bowers et al. (2016) [68] note: “Going places. Then after that I got on the paper, famous.” Some of these experiences are described by participants as different or unusual. Thus, one participant defines them as “Something different and probably once in a lifetime chance to have.” [73]. In the same study, another testimony specifically highlights the experience of travelling abroad and flying for the first time: “I went to Geneva oh I think it was 2007 oh it was brilliant… going on a plane and going abroad because I’ve never been on a plane before going abroad” [73].

The testimonies also reflect both completed trips and the desire to access new experiences of this kind. For instance, “I have gone to Almería with the team” and “I would like to go not only to Cork but also to other place” [71].

##### Transition to Adulthood

The transition to adulthood emerges as primarily associated with the development of autonomy, independence, and the progressive assumption of responsibilities. Sports practice is presented as a space that fosters confidence to act independently and make decisions about one’s own life, as reflected in the expression “I can do things by myself” [69]. This greater independence is also manifested in the possibility of carrying out activities with reduced family supervision: “Without my family worrying about what time I get home” [71]. Likewise, sport appears linked to the construction of an adult identity and the acquisition of new responsibilities: “just makes me become a man” [83]. Overall, these testimonies reflect a process of progressive autonomy and the construction of one’s own life, described as “a step toward one’s own life. A big step” [67].

##### Negative Statements and Barriers

The emerging theme negative statements and barriers brings together negative experiences and obstacles related to access, participation, and sports practice. The testimonies show that these experiences are diverse and do not respond to a single phenomenon, but rather encompass mobility and transport barriers, accessibility and support difficulties, situations of exclusion, economic inequalities, organizational issues, and the physical consequences of sports practice.

Among the main difficulties, those related to mobility and transport stand out, as they can condition the autonomy required to access sports practice. This dependence is reflected in statements such as “My dad has to bring me here. I can’t drive” [80] or in the difficulties associated with long commutes: “I have to take two buses and I get home around 11. I don’t have enough time; it’s like I have to make too many detours” [71]. Relatedly, some testimonies evidence the need to have support in order to participate: “I am always dependent on someone coming along. I can’t go out there alone anyway” [76]. Situations of exclusion or participation constraints linked to disability are also identified. Some participants describe how their opportunity to play sports was directly restricted: “They would not let me play. The only thing I could do was to carry water bottles. They were afraid I could get injured” [64], while others point out the lack of resources or supports to facilitate their inclusion: “They say they didn’t have any other disabled players and that they couldn’t help me” [64]. Added to this are financial barriers, both due to the cost of activities, “You have to pay so much, a hobby costs money” [67], and due to the perception of inequality compared to other athletes: “we do not get anything and with our own little money we need to pay a thousand Euros in order to get to play and represent Finland. In my opinion that does not sound fair” [67].

Finally, negative experiences can also occur during the sports practice itself. These include organizational difficulties and stressful situations, as shown by the testimony: “Especially in this big competition, we had a tough schedule. We had to get up early […] It was one of the most stressful moments of my life” [79], as well as injuries and physical consequences: “I went for the ball, twisted my knee and it like clicked and like I’ve got like a ligament sprain” [73]. Taken together, these testimonies show that the sports experience can be conditioned by personal, economic, organizational, and environmental obstacles that limit access, participation, or a positive experience of sports practice.

##### Having a Supportive Role

The emerging theme having a supportive role shows the importance that various support figures acquire in the participants’ sports experience. The testimonies particularly emphasize the role of coaches and instructors, whose relationship can extend beyond strictly athletic guidance. Thus, one participant describes her coach by stating: “The coach is like a second mum. A friend, a second mum […], she’s everything” [63], while another participant notes about his coach: “He is like a dad to me yeah, he tells me off sometimes and he, he wants me to succeed in life” [73]. In other cases, this relationship is expressed in terms of friendship, as shown by the statement “[Instructor name] is my friend, [Instructor name] is my best friend” [80]. Support also appears linked to accompaniment in achieving goals and improving within sports practice. Oskarsson et al. (2025) [79] explicitly capture: “I have pretty good coach support. So I can [reach my goals]. I normally train and then I talk about what I need to work on and what I can improve.” Likewise, participants describe monitoring based on communication regarding their needs prior to training: “Sometimes we check in, like, how are you feeling ahead of tonight’s training? This is the idea, will that work for you, and so on” [79]. Finally, support does not come solely from sports professionals, but also from the family and social environment. In this regard, one participant points out: “My cousins and relatives have included me and cheered me. Now that I’m here in the games, they keep their fingers crossed for me, friends and everybody” [67].

## 4. Discussion

Considering the statements made by people with IDs across the different research studies, participants’ published accounts associated sport participation with several aspects of QoL, as suggested by the international literature [8]. Furthermore, the findings of the present study indicate that participants perceived benefits across all dimensions of QoL, although the extent of these benefits varied between dimensions, in line with the findings reported by Ocete et al. (2025) [15].

The thematic synthesis reinforces the view that the benefits of sport extend beyond physical activity alone, encompassing emotional and psychological gains through the enhancement of self-confidence and autonomy, as previously reported in the literature [4,6,23]. These benefits may have a particularly important impact on the everyday lives of people with IDs, given that these are areas of life that frequently represent a source of concern for both individuals themselves and those closest to them.

The dimension of emotional well-being, together with that of interpersonal relationships, constituted relevant elements in the analysis. The testimonies analysed reflect the value that people with intellectual disabilities attribute to the social bonds generated around sports practice and the well-being they report that it provides them. Participants described sport as a relational context that facilitates the development of friendships, the establishment of meaningful social connections, and the strengthening of a sense of belonging through experiences of acceptance, recognition, and mutual support. These findings align with studies indicating that sports participation can expand opportunities for social interaction, foster new relationships, and strengthen a sense of belonging to a group or community [24,25,26,28]. In particular, McConkey et al. (2019) [24] conceptualise these experiences through the term togetherness, which integrates elements such as equality, friendship, participation, connections, and mutual support. This perspective suggests that the social dimension of sport extends beyond mere involvement in an activity, acquiring significance through the quality of interpersonal bonds and shared experiences generated within this context.

Participation in collective sporting activities also provides a favourable context for personal development—the second most frequently represented QoL dimension in participants’ accounts—and for the promotion of self-determination among people with intellectual disabilities. Sport facilitates the acquisition of new skills, strengthens confidence in one’s own abilities, and encourages the assumption of responsibilities while simultaneously creating opportunities to exercise leadership, make decisions, and pursue personal aspirations. These findings are consistent with Fraser et al. (2025) [25], who reported that participation in community sport promotes life skills, self-confidence, leadership, and other personal and social competencies, building upon the evidence previously summarised by Developmental Disability WA (2022) [27]. Likewise, Darcy et al. (2013) [17] and Eime et al. (2013) [23] found that sport participation enhances self-esteem, self-confidence, resilience, goal setting, and emotional regulation, thereby increasing the satisfaction derived from achieving personal goals.

It is worth noting that, according to the findings, sport can acquire meaningful significance for individuals with IDs by constituting a meaningful occupation of their daily time, providing them with an activity in which to be actively and continuously engaged. This finding connects with Lancioni et al. (2025) [33], who highlight the importance of individuals with IDs being able to remain constructively involved in activities for extended periods, independently of staff supervision. Likewise, the meaning attributed to sport as an activity to engage in and a way of occupying time can be framed within its function as a leisure activity. In this regard, Armila et al. (2018) [36] explicitly conceptualise the sports practice of young people with intellectual disabilities as part of their free-time activities, emphasizing the importance of having genuine opportunities to access these experiences—which provide added value to the occupation of free time for individuals with IDs—and moving beyond the view of sport purely through a competitive, rehabilitative, or therapeutic lens.

The findings also point to a certain involvement in the transition to adulthood, as the testimonies reflect experiences related to greater autonomy, decision-making, responsibility, and independence. In a similar vein, Varahra et al. (2022) [5] highlight the importance of developing skills linked to mobility, social competencies, and independent self-care during adolescence, given their potential contribution to subsequent educational and employment participation, and advocate for the incorporation of sport and exercise into transition planning. From this perspective, sports practice can constitute a relevant context for learning and exercising autonomy to meet the demands of adult life, even if the development of this process cannot be attributed to sport alone.

The results of this metasynthesis suggest that sport can constitute a space for the exercise of rights for people with intellectual disabilities through participation on equal terms, recognition, respect, and inclusion. This perspective finds support in Article 30.5 of the Convention on the Rights of Persons with Disabilities, which recognises the right to participate in sporting activities on an equal basis and in accordance with the principles of equal opportunity and non-discrimination [22].

The effective exercise of the right to sport requires moving beyond mere access to the activity to consider the conditions under which participation takes place. From this perspective, Kiuppis (2018) [29] places individual choice at the centre of sports inclusion, acknowledging the importance of enabling people with disabilities to decide, according to their preferences, how and with whom to participate, thereby linking the exercise of this right with self-determination. The analyzed accounts expand this perspective by reflecting experiences of acceptance, respect, and mutual recognition, suggesting that sport can also constitute a context for developing more egalitarian relationships. This interpretation is consistent with the literature indicating that inclusive sports programmes can foster relationships based on equality and mutual recognition [28], as well as promote inclusion processes and contribute to reducing discrimination against people with disabilities [40,41,42]. Taken together, these findings reinforce a view of sports participation based not merely on being present or having access, but on having genuine opportunities to choose, participate, and build relationships on equal terms.

However, effective exercise remains conditioned by the persistence of structural and social barriers, which contribute to lower levels of sports participation among individuals with intellectual disabilities compared to the general population [17,18,25]. Despite sport offering opportunities for inclusion and well-being, participation is not exempt from such barriers. In line with our findings, Fraser et al. (2025) [25] identified obstacles related to accessibility, transport, costs, support, and the availability of sports opportunities. The data indicate that financial barriers may prevent some individuals from enjoying sports practice and, consequently, miss the opportunity to enhance their QoL—an aspect that contrasts with the significant growth driven by various international sports organizations [34,35]. These findings suggest that the inclusive potential of sport depends, in part, on the conditions and support systems that enable effective participation for people with IDs. In this regard, public administrations can play a major role in sustaining sports activities without significant disparities across individuals.

Mention should also be made of the presence of key support figures, a theme that emerges as a salient element in the sports experience of individuals with IDs. This finding is supported by studies highlighting the influence of support professionals, coaches, family members, and peers on the opportunities and conditions for physical activity and sports participation [37,39]. Specifically, Fjellström et al. (2024) [38] emphasize the role of support staff in aspects such as communication, activity structuring, and motivation, viewing them as a crucial element in facilitating physical activity practice for people with IDs.

Another important finding concerns the limited number of scientific studies that simultaneously met the required methodological quality standards and incorporated first-person accounts from people with IDs. This remains the case despite growing efforts within the scientific community to include people with disabilities directly in research, even when doing so presents methodological challenges [57]. Consequently, the present study highlights the need to reconsider current research practices and the large proportion of published studies that continue to overlook first-person perspectives. Nevertheless, the methodological quality of the studies included provided access to rich and valuable qualitative evidence.

At the same time, the studies that did include first-person accounts consistently demonstrated sufficient methodological sensitivity to incorporate cognitive accessibility measures and appropriate support strategies, thereby enabling people with IDs to participate meaningfully in research, as recommended by Hollomotz (2018) [55]. As a result, it was relatively straightforward to identify studies that explicitly described these methodological adaptations. Although a larger body of research meeting all these criteria would undoubtedly strengthen the available evidence, it is encouraging that studies incorporating first-person perspectives are generally characterised by rigorous methodological standards, particularly regarding the cognitive accessibility of the research process.

## 5. Conclusions

The findings suggest that, from the perspective of individuals with IDs participating in the included studies, the sports experience is linked to multiple dimensions of their quality of life and acquires a meaning that transcends mere physical practice. The accounts particularly reflect experiences associated with relationships and a sense of belonging, emotional well-being, skill development, autonomy, and social participation, alongside aspects related to health and the opportunities derived from sports practice. Likewise, some testimonies position sport as a space from which to exercise and advocate for rights related to participation, equal opportunities, respect, and non-discrimination. From the voices of people with IDs themselves, sport thus emerges as a context with the potential to foster experiences related to social inclusion.

The testimonies further demonstrate that these experiences do not occur in isolation; rather, the various QoL dimensions appear interconnected and conditioned by available supports, participation opportunities, and environmental characteristics. The identification of emerging themes—and particularly those of a cross-cutting nature—reinforces this multidimensional interpretation of the sports experience. At the same time, the presence of financial, transport, accessibility, organizational, or exclusionary barriers shows that sports participation does not intrinsically constitute a positive experience in itself.

Consequently, the findings allow for an interpretation of sport as a potentially meaningful context for QoL, whose value—from the perspective of people with IDs themselves—seems to depend not only on the activity performed, but also on the relationships, supports, opportunities, and conditions that accompany their participation.

Taken together, the emerging themes identified expand the understanding of the impact of sport beyond QoL dimensions. The accounts show that sports practice can provide occupation and routine, encouraging an active lifestyle and aiding in the organization of daily life, as well as facilitating new experiences, particularly travel and participation in competitions. Elements linked to the transition to adulthood also appear, mainly associated with autonomy, independence, and the assumption of responsibilities. Concurrently, sports experiences are conditioned by barriers and negative experiences, including transport and accessibility difficulties, reliance on supports, exclusion, financial costs, organizational issues, and injuries. Faced with these challenges, key support figures acquire prominent relevance—especially coaches, instructors, family members, and other individuals within the social environment—who accompany participation and the attainment of goals.

Finally, the review notes a limited presence of studies that directly incorporate first-person accounts from individuals with IDs. This finding highlights the need to continue promoting research that directly embeds their lived experiences and perspectives, thereby reducing reliance on proxy interpretations and reinforcing their active role as primary contributors to the generation of knowledge regarding their own experiences.

## 6. Future Research and Study Limitations

The heterogeneity of the included studies must be taken into account, as the accounts stem from diverse contexts, sporting disciplines, and participation conditions. While this diversity allows for the inclusion of a broad range of experiences, it also restricts direct comparison across studies, given that the meanings assigned to sports practice may be shaped by the specific characteristics of each context and mode of involvement.

On the other hand, the analysis was conducted using direct participant quotes extracted from the primary studies. Consequently, the corpus constitutes a secondary source of qualitative data, inherently conditioned by the selection and presentation choices of the original researchers.

Furthermore, the limited consideration of reflexivity in several primary studies makes it challenging to evaluate the extent to which the relationship between researchers and participants may have influenced data generation, selection, and interpretation. This factor must be borne in mind when assessing confidence in the synthesized findings.

Finally, the analyzed accounts demonstrate a predominance of positive experiences associated with sports practice, although negative experiences and various barriers to participation were also identified. This predominance should be interpreted cautiously, as it is not possible to determine whether it reflects an overwhelmingly positive appraisal of sport by individuals with IDs or whether it is partially conditioned by sample characteristics, the objectives of the primary studies, and the decisions made regarding the selection of published quotes.

These limitations highlight, in turn, a key avenue for future research. The limited availability of studies directly capturing the first-person voice of individuals with IDs underscores the value of conducting new qualitative studies based on in-depth interviews with athletes with IDs. This would expand the available body of evidence and allow for a direct, contextualised exploration of their experiences, granting greater prominence to their own perspectives in the study of the relationship between sport and quality of life.

## Figures and Tables

**Table 1:** Studies included in the qualitative analysis process.

	Autor	País	N	Women (%)	Age	Disability	Sport	Method
1	Asunta, et al. 2022 [67]	Finland	31	29%	30 (Average)	intellectual disability	Floorball, snowshoeing, cross country skiing, alpine skiing and figure skating and other summer sports	Focus group
2	Bowers, et al. (2016) [68]	Ireland	21	–	Adults & young-adults	intellectual disability	Special Olympics Sports (Various sports)	Interview
3	Bains, et al. (2020) [69]	United Kingdom	12	33.%	23–53	intellectual disabilities	Walking, Running, Dance, Paralympics Sports… (Various sports)	Interview
4	Corazza, et al. (2017) [64]	United Kingdom & Italy	14	–	Adults	intellectual disabilities	Rugby	Focus group
5	Dailey, et al. (2020) [70]	USA	19	36.8%	31 (Average)	intellectual disability	Aquatics, basketball, soccer, softball, football, golf, gymnastics, kayaking, cycling, tennis… (Various sports)	Interview
6	Elipe-Lorenz et al. (2026) [63]	Spain	6	50%	23–28	Down syndrome	Basketball	Interview
7	Elipe-Lorenz et al. (2025) [71]	Spain	4	25%	23–28	intellectual disability	Rugby	Focus group
8	Fabris et al. (2023) [72]	Croatia	8	50%	29–50	intellectual disability	Swimming and athletics	Interview
9	Hudson et al. (2018) [73]	United Kingdom	8	12.5%	24–51	intellectual disability	football	Interview
10	Kapsalakis et al. (2024) [74]	Greece	13	30.8%	18–37	intellectual disability	Football, walk, athletics, run and others	Interview
11	Kim et al. (2025) [66]	Canada	12	58.3%	13–58 (33.3 Average)	intellectual disability	Bowling, floor hockey, swimming and downhill skiing.	Interview
12	Love et al. (2016) [75]	USA	30	60%	18–71	intellectual disability	volleyball, basketball, swimming, bowling, tennis and running.	
13	Maenhout et al. (2026) [65]	Belgium	7	57%	16–22 (18.9 average)	intellectual disability	Various sports	Interview
14	Mauro, et al. (2021) [76]	Germany	24	62.5%	21–68	intellectual disability	Various sports	Interview
15	Mitchell, et al. (2016) [77]	United Kingdom	19	-	Adults	intellectual disability	Walking	Interview and focus group
16	Olvhøj, et al. (2025) [78]	Denmark	4	100%	21–57	intellectual disability	Dance	Interview
17	Oskarsson et al. (2025) [79]	Sweden	15	53.3%	20–38	intellectual disability	Alpine skiing, athletics, basketball, cross-country skiing, floorball, football, handball, judo and swimming.	Interview
18	Savage, et al. (2023) [80]	USA	11	54.5%	27–50	intellectual disability	Fitness	Interview
19	Selander et al. (2023) [81]	Sweden (English)	7	-	adults	intellectual disability	Orienteering, running, athletics and others	Interview
20	Srinivasan, et al. (2025) [82]	USA	12	50%	19–21	intellectual disability	Walking, swimming, running, tennis and others.	focus group
21	Stokowski et al. (2020) [83]	USA	9	-	19.6 (average)	intellectual disability and learning disability	American football	Interview
22	Svanelöv et al., (2020) [84]	Sweden (English)	17	35.3%	19–51	intellectual disability	Floorball, athletics, bowling, and football.	Interview
23	Walters, (2026) [85]	New Zealand (English)	11	42,3%	20–85	intellectual disability	Athlete	Interview
24	Westrop et al. (2025) [86]	Scotland (English)	12	50%	adults	intellectual disability	Walking (physical activity)	Interview and focus group

**Table 2:** Evaluation of Critical Appraisal Skills Programme (CASP).

Criteria	Totally Met (n)	Partially Met (n)	Not Met (n)
1. Was there a clear statement of the aims of the research?	24	0	0
2. Is a qualitative methodology appropriate?	24	0	0
3. Was the research design appropriate to address the aims of the research?	24	0	0
4. Was the recruitment strategy appropriate to the aims of the research?	15	9	0
5. Were the data collected in a way that addressed the research issue?	24	0	0
6. Has the relationship between researcher and participants been adequately considered?	12	7	5
7. Have ethical issues been taken into consideration?	23	0	1
8. Was the data analysis sufficiently rigorous?	20	4	0
9. Is there a clear statement of findings?	24	0	0
10. How valuable is the research?	24	0	0

**Table 3:** Thematic synthesis of Emotional Well-being (EW).

Illustrative Quotations	Author/Page	Descriptive Themes	Analytical Themes
*“I just like to do sports, it makes me happy.”*	Walters (2026) [85]	Positive emotional experiences associated with sport	Sport provides a context for enjoyment and positive emotional experiences.
*“Have fun. Enjoy it. I’ve enjoyed every minute of it, and that’s what it’s about.”*	Walters (2026) [85]		
*“I exercise and I’m happy.”*	Savage et al. (2023) [80]		
*“I go to rugby to enjoy it to the fullest.”*	Elipe-Lorenzo et al. (2025) [71]		
*“It is fun that everybody feels accepted. It [football] is not about our deficits; instead, football is fun […]”*	Svanelöv et al. (2020) [84]	Emotional well-being through belonging, acceptance and interpersonal connection	Positive emotional experiences are intertwined with feeling accepted, supported and connected to others.
*“I love having this team; they encourage me a lot.”*	Elipe-Lorenzo et al. (2026) [63]		
*“That’s right, it makes you happy, chatting with your friends. You look forward to it.”*	Westrop et al. (2025) [86]		
*“It is a unique experience. I felt welcomed and important despite never having played rugby before.”*	Corazza et al. (2017) [64]		
*“I like to win, but winning is not everything. I also want to feel that I have done my very best and that I have developed.”*	Oskarsson et al. (2025) [79]	Emotional well-being through competence, achievement and personal growth	The emotional experience of sport is connected to perceptions of competence, achievement and personal development.
*“… but it’s so much fun, and that’s mostly why. Also, you want to grow and develop as well.”*	Oskarsson et al. (2025) [79]		
*“Playing in a team is good, it gives us confidence.”*	Elipe-Lorenzo et al. (2025) [7]		
*“Oh, I think it’s good that you can be who you are.”*	Oskarsson et al. (2025) [79]	Authenticity and positive self-perception through sport	Sport provides a context in which participants can experience authenticity and construct a more positive perception of themselves.
*“just be yourself when you step out.”*	Oskarsson et al. (2025) [79]		
*“Yeah, well, it makes you feel important. It makes you feel more…yeah yeah.”*	Hudson et al. (2018) [73]		
*“It stimulates your mind doesn’t it, playing football…you get rid of a lot of negative stuff that you think about…and makes you rethink what you’re doing.”*	Hudson et al. (2018) [73]	Sport as a means of emotional regulation and coping	Participation in sport provides a space for managing negative thoughts and modifying emotional states.
*“It’s a physical game, too, like, say you got stuff on your mind, you can take it out on the field.”*	Stokowski et al. (2020) [83]		
*“Yeah, I’ve been trying to back out, too. But then I think, just do it, it’s good for you, then you’re in a completely different mood when you get home.”*	Mauro et al. (2021) [76]		
*“It’s okay, I feel safe playing MA.”*	Elipe-Lorenzo et al. (2025) [7]	Emotional safety, care and expression within sport	A supportive sporting environment provides conditions for emotional safety, care and emotional expression. (*)
*“We can share our emotions.”*	Elipe-Lorenzo et al. (2025) [7]		
*“The MA environment is careful with me.”*	Elipe-Lorenzo et al. (2025) [7]		
*“There’s a wee park up beside me you know, so I walk there and then I walk back down. You know, and just kind of sit about a wee while and then say right. It’s not even just that, the fresh air gives you…makes you feel good.”*	Westrop et al. (2025) [86]	Positive emotional experiences associated with the physical and natural environment	The emotional experience of physical activity can extend beyond the activity itself to the environment in which participation occurs.

* Note. The quotations contributing to these analytical themes derive from studies rated as partially met for CASP criterion 6 (consideration of the relationship between the researcher and participants). Therefore, these analytical interpretations should be considered with caution, as the potential influence of the researcher on the production and interpretation of the primary data cannot be fully assessed.

**Table 4:** Thematic synthesis of interpersonal relationships (IR).

Illustrative Quotations	Author	Descriptive Themes	Analytical Themes
*“I love meeting people, getting involved with all the sports.”*	Walters (2026) [85]	Sport facilitates meeting people and expanding social relationships.	Sport provides opportunities for establishing and extending interpersonal connections.
*“I’ve got to know a lot more people [after 12 years in my sport].”*	Walters (2026) [85]		
*“I love to socialise in Special Olympics and meet new people that I do the activities with.”*	Bowers et al. (2016) [68]		
*“Seeing new people and mixing with other people.”*	Hudson et al. (2018) [73]		
*“It is good to have company. To hang out, you are not alone.”*	Fabris et al. (2023) [72]	Friendships provide companionship and opportunities for shared experiences.	Relationships developed and maintained around sport provide companionship that extends beyond participation itself.
*“I hang out with two athlete friends in my spare time.”*	Selander et al. (2023) [81]		
*“Getting out and relaxing with my friends…I made friends through football as well…some of them are funny and some of them are loud.”*	Hudson et al. (2018) [73]		
*“I walk with my friend in class, we love TRX.”*	Savage et al. (2023) [80]		
*“So that you can confide in him, have a shoulder to cry on, cheer you up when you are sad, hang out, tell a secret and so on.”*	Fabris et al. (2023) [72]	Interpersonal relationships provide emotional and practical support.	Sport-related relationships can develop into meaningful sources of mutual support and care.
*“The best thing is that you meet people, play basketball, get a bit more motivated, and teammates always give you a hand.”*	Elipe-Lorenzo et al. (2026) [63]		
*“I love having this team; they encourage me a lot.”*	Elipe-Lorenzo et al. (2026) [63]		
*“My cousins and relatives have included me and cheered me. Now that I’m here in the games, they keep their fingers crossed for me, friends and everybody.”*	Asunta et al. (2022) [67]		
*“The coach is like a second mum. A friend, a second mum […], she’s everything.”*	Elipe-Lorenzo et al. (2026) [63]	Coaches and instructors can be experienced as significant supportive relationships.	Interpersonal relationships in sport extend beyond peers, with coaches and instructors becoming meaningful support figures.
*“I have pretty good coach support. So I can [reach my goals]. I normally train and then I talk about what I need to work on and what I can improve.”*	Oskarsson et al. (2025) [79]		
*“[Instructor name] is my friend (P05), [Instructor name] is my best friend.”*	Savage et al. (2023) [80]		
*“The family we have formed together. It is very beautiful.”*	Elipe-Lorenzo et al. (2026) [63]	Team relationships are described through a sense of unity, familiarity and family-like bonds.	Shared sporting participation can transform team membership into close relational bonds characterised by unity and familiarity.
*“Everyone is friends with each other—we spend time together during training and have this sense of familiarity with what most others are saying.”*	Oskarsson et al. (2025) [79]		
*“It’s like a family, you become like a family.”*	Stokowski et al., (2020) [83]		
*“Even playing with the rival, it also feels like we are a big family, which is rugby.”*	Elipe-Lorenzo et al. (2025) [7]		
*“Proper respect. You finish the match and you say well done team, you played well.”*	Hudson et al. (2018) [73]	Relationships in sport involve reciprocity, respect and helping others.	Sport provides a relational context in which participants can both receive and provide support, encouragement and recognition.
*“I try to lead and also help out the other lads as well.”*	Hudson et al. (2018) [73]		
*“Being nice to the young new athletes.”*	Bowers et al. (2016) [68]		
*“and that everyone is cheering each other.”*	Oskarsson et al. (2025) [79]		
*“We always have a laugh at football it’s quite… it’s good it’s a good laugh. The banter is good.”*	Hudson et al. (2018) [73]	Humour and shared enjoyment form part of interpersonal interaction in sport.	Everyday social interactions, including humour and shared enjoyment, contribute to the relational experience of sport.
*“I enjoy that. When you’re there with others, you can have a good laugh.”*	Mauro et al. (2021) [76]		
*“That’s right, it makes you happy, chatting with your friends. You look forward to it.”*	Westrop et al. (2025) [86]		
*“because when I am out I have to interact with people, so it has helped me with that… It forces me to talk to other people when I wouldn’t have talked to them before.”*	Mitchell et al. (2018) [77]	Sport creates opportunities to practise social interaction and communication.	Participation can provide a context for developing interpersonal engagement by creating opportunities for communication with others.
*“Well, it is a way to get to know new people. I try to include others […] I am shy. I try to get over it, but it takes time. I find it hard to meet new people. But all people are nice.”*	Svanelöv et al. (2020) [84]		
*“I like being with the whole team to chat, to build the team, build unity.”*	Elipe-Lorenzo et al. (2026) [63]		

**Table 5:** Thematic synthesis of personal development (PD).

Illustrative Quotations	Author	Descriptive Themes	Analytical Themes
*“It takes time to learn new things. It always does. When we learn new things in floorball, we take our time—in peace and quiet—so everybody can keep up. It [activities and training sessions] takes longer but nobody gets stressed that you should be able to know it fast.”*	Svanelöv et al. (2020) [84]	Learning and development of new skills through sport	Sport provides opportunities for learning and progressive skill development.
*“When I discovered [downhill skiing] competitively like a few years ago, I was really good and I’ve already had the fundamentals down.”*	Kim et al. (2025) [66]		
*“And now, step by step, with my acquired new skills and abilities, I’m learning to realise that I can do more things than I thought.”*	Corazza et al. (2017) [64]		
*“I like to win, but winning is not everything. I also want to feel that I have done my very best and that I have developed.”*	Oskarsson et al. (2025) [79]	Personal growth and improvement through sporting participation	Development is experienced not only through outcomes, but through perceptions of progress and becoming more capable.
*“… but it’s so much fun, and that’s mostly why. Also, you want to grow and develop as well.”*	Oskarsson et al. (2025) [79]		
*“You grow as a person, and we aren’t so fragile. We’re stronger than others think.”*	Selander et al. (2023) [81]		
*“The best part of sport is that it gives selfconfidence. You can do stuff.”*	Selander et al. (2023) [81]	Development of confidence and perceived capability	Sporting experiences contribute to a developing sense of capability and confidence in one’s own abilities.
*“I can perform the activity and feel competent.”*	Oskarsson et al., (2025) [79]		
*“It means I’m being confident for myself … I can do things by myself …. Like I’m-I’m fifty three years of age now … I don’t need much people supporting me to do that. I do it myself.”*	Bains et al., (2020) [69]		
*“[Competing at the World Games] was a life achievement for me … I opened the Games, I lead all the team in!”*	Walters (2026) [85]	Achievement and accomplishment as experiences of personal development	Sporting achievements provide experiences through which participants recognise their own progress and accomplishments.
*“It was a big achievement for me.”*	Bowers et al. (2016) [68]		
*“But the real win for me is that if I do my sport faster than I’ve ever done before, if I set a personal best and stuff like that, that’s the real victory for me. The medals matter less.”*	Oskarsson et al. (2025) [79]		
*“I would like to train two days, more than just Wednesday […]. Because we just play for one hour and we need to play more.”*	Elipe-Lorenzo et al. (2026) [63]	Motivation to progress and pursue personal sporting goals	Personal development is expressed through aspirations for continued participation, challenge and improvement.
*“I want to be challenged in the activity. Do not set limits on my development. Listen to my goals and support me to understand how to reach them.”*	Oskarsson et al. (2025) [79]		
*“My goal is to win a gold medal at an international championship, where I compete in my sport.”*	Oskarsson et al. (2025) [79]		
*“was glad I joined Special Olympics ‘cause I was out of my comfort zone and then I got in my comfort zone with Special Olympics.”*	Bowers et al. (2016) [68]	Personal development through facing challenges and extending previous boundaries	Sport provides experiences of challenge through which participants describe extending their perceived capabilities.
*“I was shy at first going to different places, meeting everyone else, but now we just go everywhere, it’s a bonus now, we just get on with it and it’s alright now.”*	Hudson et al. (2018) [73]		
*“I want to be challenged in the activity. Do not set limits on my development. Listen to my goals and support me to understand how to reach them.”*	Oskarsson et al. (2025) [79]		
*“Every time we’re on a walk then what that does is then that gives me the responsibility that as a walk leader that gives me the responsibility for like everyone …”*	Westrop et al. (2025) [86]	Responsibility, leadership and supporting others	Personal development extends beyond individual competence to assuming responsibility and supportive roles towards others.
*“I try to lead and also help out the other lads as well.”*	Hudson et al. (2018) [73]		
*“I am or I want to be a role model to others,”*	Oskarsson et al. (2025) [79]		
*“because when I am out I have to interact with people, so it has helped me with that… It forces me to talk to other people when I wouldn’t have talked to them before.”*	Mitchell et al. (2018) [77]	Development of interpersonal and communication capabilities	Some developmental experiences acquired through sport extend into social interaction and engagement with others.
*“It’s there to help people with an intellectual disability to come out and express how they feel and it’s opened my eyes to the bigger picture and don’t be scared to speak about it.”*	Walters (2026) [85]		

**Table 6:** Thematic synthesis of physical well-being (PW).

Illustrative Quotations	Author	Descriptive Themes	Analytical Themes
“It’s great, I’m doing really well. I’m healthy!”	Kapsalakis et al. (2024) [74]	Health and physical well-being	Sport and physical activity are perceived as contributing to health and physical well-being.
“It’s good for your bones.”	Love et al. (2016) [75]		
“helped me keep my health in check and be more healthy [sic].”	Dailey et al. (2020) [70]		
“Sport has made me strong.”	Selander et al. (2023) [81]	Fitness, strength and physical capacity	Sport contributes to perceived fitness, strength and capacity to perform everyday activities.
“I enjoy training because it keeps me fit.”	Hudson et al. (2018) [73]		
“My training makes me feel good both socially and physically.”	Oskarsson et al. (2025) [79]		
“I want to lose weight […] I’d really like to get in shape.”	Kapsalakis et al. (2024) [74]	Weight and body-related goals	Physical activity is associated with weight management and body-related goals.
“I’ve lost weight. So we have kept to our regime, our pedometers and our belts.”	Mitchell et al. (2018) [77]		
“I’d go to the gym 3 times a week. And that would keep me active.”	Bowers et al. (2016) [68]	Active lifestyle	Physical activity supports an active lifestyle and the incorporation of movement into everyday routines.
“Since going out places I have got used to walking a lot more and I am not so out of breath.”	Mitchell et al. (2018) [77]		

**Table 7:** Thematic synthesis of Rights (RI).

Illustrative Quotations	Author	Descriptive Themes	Analytical Themes
“Everyone has the right to play or train.”	Svanelöv et al., (2020) [84]	Right to participation and opportunities	Sport is understood as a right and as an opportunity for participation.
“be a right and involve participation in society, that is what matters most.”	Elipe-Lorenzo et al., (2026) [63]		
“They give me the opportunity.”	Elipe-Lorenzo et al., (2025) [71]		
“No one is judged for their disability—there is no ‘us’ or ‘them’.”	Oskarsson et al., (2025) [79]	Equality, respect and non-discrimination	Sporting environments can provide experiences of equal treatment, respect and non-discrimination.
“The environment respects us.”	Elipe-Lorenzo et al., (2025) [71]		
“They would not let me play. The only thing I could do was to carry water bottles. They were afraid I could get injured.”	Corazza et al., (2017) [64]	Barriers and support for participation	Participation can be restricted by exclusion, while appropriate support facilitates involvement.
“What I’m passionate about is making sure that everyone is seen and receives help, without judging anyone because of their disability.”	Oskarsson et al., (2025) [79]		
“‘If you have a disability, you must be helped out, not be left alone.”	Asunta et al., (2022) [67]		

**Table 8:** Thematic synthesis of material well-being (MW).

Illustrative Quotations	Author	Descriptive Themes	Analytical Themes
*“But if there was the possibility, to get paid for doing your sport, it would have been priceless for me. Since, then, you could spend much more time on sport. You could spend a lot more time on individual workouts.”*	Selander et al. (2023) [81]	Economic conditions influence the possibility of dedicating more time to sport.	Material and employment circumstances shape the possibilities for sustained engagement in sport. (*)
*“If I could do sport without having to work, that would be…If I could live off sport, then I would do it. But, as it is now, it’s not possible. That’s how it is now.”*	Selander et al. (2023) [81]		
*“Thanks to Rugby they have found me a job.”*	Elipe-Lorenzo et al. (2025) [71]	Sport is associated with access to employment opportunities.	Sport can intersect with material well-being through opportunities extending beyond sporting participation. (*)
*“I have gone to Almería with the team.”*	Elipe-Lorenzo et al. (2025) [71]	Sport provides opportunities for travel and experiences outside the usual environment.	Participation in sport can broaden access to experiences and opportunities that have a material dimension. (*)
*“I would like to go not only to Cork but also to other place.”*	Elipe-Lorenzo et al. (2025) [71]		
*“I feel like I belong here, we also get these really nice clothes when going on these trips.”*	Asunta et al. (2022) [67]		
*“My goal is to win a gold medal at an international championship, where I compete in my sport.”*	Oskarsson et al. (2025) [79]	Material or symbolic recognition is expressed through sporting achievement.	Sporting participation provides access to tangible and symbolic forms of recognition.

* Note: The quotations contributing to these analytical themes derive from studies rated as partially met for CASP criterion 6 (consideration of the relationship between the researcher and participants). Therefore, these analytical interpretations should be considered with caution, as the potential influence of the researcher on the production and interpretation of the primary data cannot be fully assessed.

**Table 9:** Thematic synthesis of self-determination (SD).

Illustrative Quotations	Author	Descriptive Themes	Analytical Themes
*“To be able to choose what you want to do, when to do it and with whom.”*	Kapsalakis et al., (2024) [74]	Choice, decision-making and independence	Sport provides opportunities to exercise choice, make decisions and experience independence.
*“Yes, I make my decisions.”*	Elipe-Lorenzo et al., (2025) [7]		
*“We talk among ourselves and come to a joint decision.”*	Elipe-Lorenzo et al., (2025) [71]		
*“The power of the Special Olympics movement is it shows them a new way of how to be themselves but also to be independent.”*	Walters (2026) [85]		
*“A competitor. Ambitious to achieve whatever we want.”*	Elipe-Lorenzo et al., (2026) [63]	Personal goals and achievement	Sport provides a context for defining and pursuing personal goals and aspirations.
*“I want to be the best player I can be, given my circumstances. I have even dreamed of… I don’t know if it’s possible but to play in [an international championship in their sport].”*	Oskarsson et al., (2025) [79]		
*“I also want to feel that I have done my very best and that I have developed.”*	Oskarsson et al., (2025) [79]		
*“I am a brave girl. I won’t ever give up; I keep fighting, and do it over and over again.”*	Olvhoj et al., (2025) [78]	Effort and persistence	Sport is associated with effort, perseverance and continuing in the face of difficulty.
*“It taught me how to work hard, like, I know I’m lazy, but then again I’m not.”*	Stokowski et al., (2020) [83]		
*“I know it’s hard…trying your hardest to win…win the cup… it is hard.”*	Hudson et al., (2018) [73]		
*“Every time we’re on a walk then what that does is then that gives me the responsibility that as a walk leader that gives me the responsibility for like everyone …”*	Westrop et al., (2025) [86]	Responsibility and active involvement	Sport provides opportunities to assume responsibility and take an active role in sporting activities.
*“It’s part of me. It’s part of what I owe – I owe it to the coaches and team – no skiving off. I owe it to them to be there to help in tournaments.”*	Hudson et al., (2018) [73]		
*“And I say, please can we practice it.”*	Elipe-Lorenzo et al., (2025) [7]		

**Table 10:** Thematic synthesis of Social Inclusion (SI).

Illustrative Quotations	Author	Descriptive Themes	Analytical Themes
*“One of the most important aspects is the feeling of community, a sense of shared joy and familiarity with others.”*	Oskarsson et al., (2025) [79]	Belonging, inclusion and acceptance	Sport fosters belonging and inclusion by providing a context in which participants feel accepted and part of a community.
*“a sense of belonging. The team is like a new family.”*	Selander et al., (2023) [81]		
*“There is inclusion if we all get together, if we separate it is not the same.”*	Elipe-Lorenzo et al., (2025) [71]		
*“I have Asperger Syndrome. It’s hard for me to communicate but now I’m part of a team and socialise in the bar after training and games.”*	Corazza et al., (2017) [64]	Social interaction and relationships	Sport creates opportunities for social interaction and developing connections with others.
*“Everyone is friends with each other—we spend time together during training and have this sense of familiarity with what most others are saying.”*	Oskarsson et al., (2025) [79]		
*“My club is about community, where everyone gets to be involved,”*	Oskarsson et al., (2025) [79]	Participation and involvement	Sport provides opportunities for active involvement and participation as part of a group.
*“stumbled upon this same thing at school. I was always left aside. Now I’m with the group!”*	Asunta et al., (2022) [67]		

## Data Availability

The raw data supporting the conclusions of this article will be made available by the authors on request.

## Supplementary Materials

The following supporting information can be downloaded at: https://www.mdpi.com/article/10.3390/healthcare14183089/s1.

## Abbreviations

The following abbreviations are used in this manuscript:

QoL	Quality of Life
IDs	Intellectual Disabilities

## References

[1] Bouzas S., Martínez-Lemos I., Ayán C. (2019). Effects of exercise on the physical fitness level of adults with intellectual disability: A systematic review. Disabil. Rehabil..

[2] World Health Organization (2022). Global Status Report on Physical Activity 2022.

[3] Duijvestijn M., de Wit G.A., van Gils P.F., Wendel-Vos G.C.W. (2023). Impact of physical activity on healthcare costs: A systematic review. BMC Health Serv. Res..

[4] Kapsal N.J., Dicke T., Morin A.J.S., Vasconcellos D., Maïano C., Lee J., Lonsdale C. (2019). Effects of physical activity on the physical and psychosocial health of youth with intellectual disabilities: A systematic review and meta-analysis. J. Phys. Act. Health.

[5] Varahra A., Ahmed H., Lindsay S. (2022). Exploring direct and indirect associations of exercise and sport participation with employment among individuals with disabilities: A scoping review. J. Occup. Rehabil..

[6] Borland R.L., Cameron L.A., Tonge B.J., Gray K.M. (2022). Effects of physical activity on behaviour and emotional problems, mental health and psychosocial well-being in children and adolescents with intellectual disability: A systematic review. J. Appl. Res. Intellect. Disabil..

[7] Elipe-Lorenzo P., Diez-Fernández P., Ruibal-Lista B., López-García S. (2025). Barriers Faced by People with Disabilities in Mainstream Sports: A Systematic Review. Front. Sports Act. Living.

[8] Franco E., Ocete C., Pérez-Calzado E., Berástegui A. (2023). Physical activity and quality of life among people with intellectual disabilities: The role of gender and the practice characteristics. Behav. Sci..

[9] Bondár R.Z., Di Fronso S., Bortoli L., Robazza C., Metsios G.S., Bertollo M. (2019). The effects of physical activity or sport-based interventions on psychological factors in adults with intellectual disabilities: A systematic review. J. Intellect. Disabil. Res..

[10] Thomas J., Harden A. (2008). Methods for the thematic synthesis of qualitative research in systematic reviews. BMC Med. Res. Methodol..

[11] Verdugo M.A., Schalock R.L., Keith K.D., Stancliffe R.J. (2005). Quality of life and its measurement: Important principles and guidelines. J. Intellect. Disabil. Res..

[12] Verdugo M.Á., Schalock R.L. (2010). Últimos avances en el enfoque y concepción de las personas con discapacidad intelectual. Siglo Cero.

[13] Verdugo M.Á., Schalock R.L., Gómez Sánchez L.E. (2021). The Quality of Life Supports Model: Twenty-five years of parallel paths have come together. Siglo Cero.

[14] Gómez L.E., Schalock R.L., Verdugo M.Á. (2021). A new paradigm in the field of intellectual and developmental disabilities: Charac- teristics and evaluation. Psicothema.

[15] Ocete C., Rocuant-Urzúa A., Fernández-Rivas M., Franco E. (2025). Do people with intellectual disabilities have a better quality of life if they are physically active?. Eur. J. Investig. Health Psychol. Educ..

[16] Rimmer J.H., Marques A.C. (2012). Physical activity for people with disabilities. Lancet.

[17] Darcy S., Dowse L. (2013). In search of a level playing field: The constraints and benefits of sport participation for people with intel- lectual disability. Disabil. Soc..

[18] Taheri A., Perry A., Minnes P. (2016). Examining the social participation of children and adolescents with intellectual disabilities and autism spectrum disorder in relation to peers. J. Intellect. Disabil. Res..

[19] Martin Ginis K.A., van der Ploeg H.P., Foster C., Lai B., McBride C.B., Ng K., Pratt M., Shirazipour C.H., Smith B., Vásquez P.M. (2021). Participation of people living with disabilities in physical activity: A global perspective. Lancet.

[20] Simón-Siles S., Font-Farré M., Guerra-Balic M., Nishishinya-Aquino M.B., Oviedo G.R. (2022). Effects of exercise on fitness in adults with intellectual disability: A protocol of an overview of systematic reviews. BMJ Open.

[21] United Nations (2006). Convention on the Rights of Persons with Disabilities.

[22] Prieto J., Paramio-Salcines J.L. (2018). The United Nations Convention on the Rights of Persons with Disabilities and its effects on the promotion of elite disability sport: A worldwide analysis. Age Hum. Rights J..

[23] Eime R.M., Young J.A., Harvey J.T., Charity M.J., Payne W.R. (2013). A systematic review of the psychological and social benefits of participation in sport for children and adolescents: Informing development of a conceptual model of health through sport. Int. J. Behav. Nutr. Phys. Act..

[24] McConkey R., Peng C., Merritt M., Shellard A. (2019). The meaning of social inclusion to players with and without intellectual disa- bility in Unified Sports teams. Inclusion.

[25] Fraser K., Clayden M., Piggott B., Picknoll D. (2025). Benefits and barriers of participating in community sport for individuals with an intellectual disability. J. Intellect. Disabil..

[26] Zhao W.M., Thirumal K., Renwick R., DuBois D. (2021). Belonging through sport participation for young adults with intellectual 522 and developmental disabilities: A scoping review. J. Appl. Res. Intellect. Disabil..

[27] Developmental Disability WA (2022). Intellectual Disability.

[28] McConkey R., Dowling S., Hassan D., Menke S. (2013). Promoting social inclusion through Unified Sports for youth with intellectual disabilities: A five-nation study. J. Intellect. Disabil. Res..

[29] Kiuppis F. (2018). Inclusion in sport: Disability and participation. Sport Soc..

[30] Cegarra-Dueñas B., Ribes Martínez-Márquez A., Saurí Ruiz J. (2026). Barreras y facilitadores para la participación en la sociedad de las personas con discapacidad en España. Aposta Rev. Cienc. Soc..

[31] Jacinto M., Sousa A., Palmeira D., Antunes R., Matos R., Ferreira J. (2021). Perceived Barriers of Physical Activity Participation in Individuals with Intellectual Disability—A Systematic Review. Healthcare.

[32] Michalsen H., Henriksen A., Hartvigtsen G., Olsen M.I., Pedersen E.R., Søndenaa E., Jahnsen R.B., Anke A. (2024). Barriers to physical activity participation for adults with intellectual disability: A cross-sectional study. J. Appl. Res. Intellect. Disabil..

[33] Lancioni G.E., Alberti G., Filippini C., Singh N.N., O’Reilly M.F., Sigafoos J. (2025). A Technology-Aided Program to Help People With Intellectual and Multiple Disabilities Access Leisure Stimuli and Engage in Cognitive and Physical Activity: Development and Usability Study. JMIR Rehabil. Assist. Technol..

[34] Lloyd M., Temple V.A., Foley J.T., Yeatman S., Lunsky Y., Huang A., Balogh R. (2022). Young adults with intellectual and devel- opmental disabilities who participate in Special Olympics are less likely to be diagnosed with depression. Soc. Psychiatry Psychiatr. Epidemiol..

[35] Crawford C., Burns J., Fernie B.A. (2015). Psychosocial impact of involvement in the Special Olympics. Res. Dev. Disabil..

[36] Armila P., Rannikko A., Torvinen P. (2018). Young people with intellectual disabilities and sport as a leisure activity: Notions from the Finnish welfare society. Leis. Stud..

[37] Sakalidis K.E., Fadeeva A., Hettinga F.J., Ling F.C.M. (2023). The role of the social environment in inclusive sports participation—Identifying similarities and challenges in athletes with and without intellectual disabilities through coaches’ eyes: A qualitative inquiry. PLoS ONE.

[38] Fjellström S., Hölttä J., Nordström A., Flygare Wallén E., Lund Ohlsson M., Hansen E. (2024). Increasing physical activity through an adapted web-based exercise program for people with intellectual disabilities: Support staff are crucial for feasibility. J. Appl. Res. Intellect. Disabil..

[39] Bossink L.W.M., van der Putten A.A.J., Vlaskamp C. (2020). Physical-activity support for people with intellectual disabilities: A theory-informed qualitative study exploring the direct support professionals’ perspective. Disabil. Rehabil..

[40] Crespo-Eguilaz N., Gambra L., Varela A., Fraguela-Vale R. (2024). Satisfying basic psychological needs through a recreational sports programme for people with intellectual disability: Human growth and adapted sport in focus. Front. Psychol..

[41] Eather N., Wade L., Pankowiak A., Eime R. (2023). The impact of sports participation on mental health and social outcomes in adults: A systematic review and the “Mental Health through Sport” conceptual model. Syst. Rev..

[42] Scifo L., Chicau Borrego C., Monteiro D., Matosic D., Feka K., Bianco A., Alesi M. (2019). Sport intervention programs (SIPs) to improve health and social inclusion in people with intellectual disabilities: A systematic review. J. Funct. Morphol. Kinesiol..

[43] Diz S., Jacinto M., Costa A., Monteiro D., Matos R., Antunes R. (2024). Physical activity, quality of life and well-being in individuals with intellectual and developmental disability. Healthcare.

[44] Jacob U., Pillay J., Johnson E., Omoya O., Adedokun A.P. (2023). A systematic review of physical activity: Benefits and needs for maintenance of quality of life among adults with intellectual disability. Front. Sports Act. Living.

[45] Keramat S.A., Ahammed B., Mohammed A., Seidu A.A., Farjana F., Hashmi R., Ahmad K., Haque R., Ahmed S., Ali M.A. (2022). Disability, physical activity, and health-related quality of life in Australian adults: An investigation using 19 waves of a longitudinal cohort. PLoS ONE.

[46] Grabowska I., Antczak R., Zwierzchowski J., Panek T. (2022). How to measure multidimensional quality of life of persons with disabilities in public policies: A case of Poland. Arch. Public Health.

[47] Schalock R.L., Keith K.D., Verdugo M.Á., Gómez L.E., Kober R. (2011). Quality of life model development and use in the field of intellectual disability. Enhancing the Quality of Life of People with Intellectual Disabilities: From Theory to Practice.

[48] Van Hecke N., Claes C., Vanderplasschen W., De Maeyer J., De Witte N., Vandevelde S. (2018). Conceptualisation and measurement of quality of life based on Schalock and Verdugo’s model: A cross-disciplinary review of the literature. Soc. Indic. Res..

[49] Verdugo M.Á., Schalock R.L., Gómez L.E. (2023). The Quality of Life Supports Model as a major component in applying the quality of life paradigm. J. Policy Pract. Intellect. Disabil..

[50] Álvarez-Aguado I., Vega V., Farhang M., Álvarez González L., Spencer H. (2023). Quality of life of elderly people with severe intel- lectual disabilities in Chile. Rev. Cienc. Salud.

[51] Mora Antó A., Salamanca Duque L.M., Córdoba Andrade L., Gómez Sánchez L.E. (2020). Estructura dimensional de la Escala Kid- sLife, versión Colombia, para la evaluación de calidad de vida en discapacidad intelectual. Psykhe.

[52] Simões C., Santos S., Biscaia R. (2016). Validation of the Portuguese version of the Personal Outcomes Scale. Int. J. Clin. Health Psy-Chol..

[53] Wehmeyer M.L. (2020). The importance of self-determination to the quality of life of people with intellectual disability: A perspective. Int. J. Environ. Res. Public Health.

[54] Fricker M. (2007). Epistemic Injustice: Power and the Ethics of Knowing.

[55] Hollomotz A. (2018). Successful interviews with people with intellectual disability. Qual. Res..

[56] Kooijmans R., Mercera G., Langdon P., Moonen X. (2022). The adaptation of self-report measures to the needs of people with intel- lectual disabilities: A systematic review. Clin. Psychol. Sci. Pract..

[57] Samways B., Heslop P. (2025). Learning to listen to non-spoken voices. Including young people with intellectual disabilities who are non-speaking as primary participants in qualitative research. Int. J. Qual. Methods.

[58] O’Brien P., McConkey R., García-Iriarte E. (2014). Co-researching with people who have intellectual disabilities: Insights from a na- tional survey. J. Appl. Res. Intellect. Disabil..

[59] Bigby C., Frawley P., Ramcharan P. (2014). Conceptualizing inclusive research with people with intellectual disability. J. Appl. Res. Intellect. Disabil..

[60] Kaplowitz M.D., Hoehn J.P. (2001). Do focus groups and individual interviews reveal the same information for natural resource val- uation?. Ecol. Econ..

[61] Boateng W. (2012). Evaluating the efficacy of focus group discussion (FGD) in qualitative social research. J. Res. Peace Gend. Dev..

[62] Hsieh H.-F., Shannon S.E. (2005). Three approaches to qualitative content analysis. Qual. Health Res..

[63] Elipe-Lorenzo P., Resende R., Diez-Fernández P., Gonzalo Ó., Ruibal-Lista B., Coca A., da-Silva C., López-García S. (2026). “I’m not a volunteer; I’m an equal player”: The social factor of quality of life in mixed ability basketball players. Front. Sports Act. Living.

[64] Corazza M., Dyer J. (2017). A new model for inclusive sports? An evaluation of participants’ experiences of Mixed Ability Rugby. Soc. Incl..

[65] Maenhout L., Compernolle S., Cardon G., De Paepe A., Van Hove G., Crombez G. (2026). Using a theory-based approach to evaluate a physical activity promotion intervention for youth with intellectual disabilities. Soc. Sci. Med..

[66] Kim J., Ellard C., Tamminen K.A., Arbour-Nicitopoulos K.P. (2025). Special Olympics athletes’ experiences of mental health and help-seeking. J. Appl. Sport Psychol..

[67] Asunta P., Hasanen E., Kiuppis F., Rintala P., McConkey R. (2022). ‘Life is team play’: Social inclusion of people with intellectual disabilities in the context of Special Olympics. Sport Soc..

[68] Bowers K., Corby D., Lambert V., Staines A., McVeigh T., McKeon M., Hoey E., Belton S., Meegan S., Walsh D. (2016). People with intellectual disability and their families’ perspectives of Special Olympics Ireland: Qualitative findings from the SOPHIE study. J. Intellect. Disabil..

[69] Bains K.K., Turnbull T. (2020). Using a theoretically driven approach with adults with mild-moderate intellectual disabilities and carers to understand and improve uptake of healthy eating and physical activity. Obes. Med..

[70] Dailey S.L., Alabere R.O., Michalski J.E., Brown C.I. (2020). Sports experiences as anticipatory socialization: How does communication in sports help individuals with intellectual disabilities learn about and adapt to work?. Commun. Q..

[71] Elipe-Lorenzo P., Verdugo M.Á., Diez-Fernández P., Ruibal-Lista B., López-García S. (2025). “Give me the opportunity”: Mixed Ability sports and quality of life in people with intellectual disabilities. Sports.

[72] Fabris A., Bratković D., Ralić A.Ž. (2023). Self-assessment of friendships and social inclusion of adults with intellectual disabilities. Spec. Educ. Rehabil..

[73] Hudson N.A., Mrozik J.H., White R., Northend K., Moore S., Lister K., Rayner K. (2018). Community football teams for people with intellectual disabilities in secure settings: “They take you off the ward, it was like a nice day, and then you get like medals at the end”. J. Appl. Res. Intellect. Disabil..

[74] Kapsalakis P., Nteropoulou-Nterou E. (2024). Perspectives of adults with intellectual disabilities on quality of life: A qualitative study. Int. J. Environ. Res. Public Health.

[75] Love A., Agiovlasitis S. (2016). How do adults with Down syndrome perceive physical activity?. Adapt. Phys. Act. Q..

[76] Mauro A., Bruland D., Latteck Ä.-D. (2021). “With enthusiasm and energy throughout the day”: Promoting a physically active lifestyle in people with intellectual disability by using a participatory approach. Int. J. Environ. Res. Public Health.

[77] Mitchell F., Stalker K., Matthews L., Mutrie N., Melling C., McConnachie A., Murray H., Melville C. (2018). A qualitative exploration of participants’ experiences of taking part in a walking programme: Perceived benefits, barriers, choices and use of intervention resources. J. Appl. Res. Intellect. Disabil..

[78] Olvhøj R., Jensen T.K., Wienecke J., Winther H. (2025). Everyone dances the way they can: Opportunities and challenges in inclusive movement activities for adults with intellectual disability. Res. Dance Educ..

[79] Oskarsson J., Flygare Wallén E., Wickman K., Lund Ohlsson M. (2025). Empower our growth as athletes: Voices of Swedish athletes with intellectual disability. J. Appl. Res. Intellect. Disabil..

[80] Savage M.N., Colombo-Dougovito A.M. (2023). Capabilities, opportunities, and motivation: Exploring fitness program experiences of adults with intellectual and developmental disabilities. Int. J. Environ. Res. Public Health.

[81] Selander J., Wall E. (2023). The athletic work force: Sport as a key to employment for people with intellectual disabilities?. Work.

[82] Srinivasan S., Sattar N., Athreya A., Glenney S.S., Bubela D. (2025). Stakeholder perspectives on physical activity in youth with developmental disabilities: A mixed methods study. Intellect. Dev. Disabil..

[83] Stokowski S., Goldsmith A., Croft C., Hutchens S., Fridley A. (2020). The impact of football on student-athletes with education-impacting disabilities. J. Study Sports Athl. Educ..

[84] Svanelöv E., Flygare Wallén E., Enarsson P., Stier J. (2020). “Everybody with disability should be included”: A qualitative interview study of athletes’ experiences of disability sports participation analysed with ideas of able-mindedness. Scand. J. Disabil. Res..

[85] Walters T. (2026). “We’re all great athletes!” The Special Olympics social world and the creation of an athlete identity. World Leis. J..

[86] Westrop S.C., Niven A., Melville C., Speir D.-M., McGarty A.M. (2025). Understanding capabilities, opportunities, and motivations of walking for physical activity among adults with intellectual disabilities: A qualitative theory-based study. J. Appl. Res. Intellect. Disabil..

[87] CASP (2013). CASP Qualitative Research Checklist.

