# Supplementary material for: Quality of Life and Psychosocial Impact of Sport on People with Intellectual Disabilities: The Value of First-Person Discourse
Source: Healthcare (Basel). 2026 Sep 19;14(18):3089. doi: 10.3390/healthcare14183089 (PMC13606486; doi:10.3390/healthcare14183089)
Supplement: Supplementary file 1 [file healthcare-14-03089-s001.zip › File S1.pdf]

Search strategies and eligibility criteria

SEARCH STRATEGIES

The following criteria were used for the different searches, in addition to the Boolean search strategy detailed in the manuscript: ("Intellectual disability" OR "developmental disability" OR "learning disability") AND (Sport OR exercise OR "physical activity") AND (Interview OR narrative OR "focus group*" OR "qualitative research" OR "lived experience") AND adult*

WOS
Publication years (2026-2016), Document types (article), languages (english).

SCOPUS
Year - range (2016-2026), document type (limit: article), language (english), publication stage (limit to: final).

EBSCO DISCOVERY SERVICE:
Resource type: apply filters: (articles); creation date: refine: (2016–2026); language: (English).

OTHER SOURCES
The group of articles classified as “other sources” comprises studies that the authors were aware of before starting the research and that met the same criteria as the other articles.

ELIGIBILITY CRITERIA

AGE
For the age selection, we considered that participants should be over 18 years of age. As described in the article, there were some exceptions, but it was considered appropriate to include them because they met several criteria: the accounts provided related to topics concerning adults, adults represented more than 70% of the group, and the mean age was also above 18 years. In addition, in the case of Kim et al. (2025), only 2 of the 12 participants were adults, and the mean age was 33.3 years. In the case of Maenhot et al. (2026), only 2 of the 7 participants were adults, and the mean age was 18.9 years. Furthermore, in the latter case, the authors explicitly state in the study that the inclusion of two participants under 18 years of age was an exception and that the research was designed for adults.

All these criteria were considered sufficient to include the studies.

SPORTS PARTICIPATION
We considered whether the fundamental basis of the research was related to sport and whether it did not describe an activity that was exclusively rehabilitative.

PSYCHOSOCIAL APPROACH
We considered whether the studies focused on psychosocial aspects and, therefore, were not primarily centred on biomedical aspects.

INTELLECTUAL DISABILITY
We considered whether the research included people with intellectual disabilities.

AVAILABILITY OF FIRST-PERSON QUOTATIONS
We considered whether the article analysed included verbatim quotations from people with intellectual disabilities.
